# Functional Characterization of Glycosyltransferases from *Saussurea involucrata* Involved in Rutin Biosynthesis

**DOI:** 10.3390/ijms27219660

**Published:** 2026-10-28

**Authors:** Peiqin Yi, Bingying Huang, Ruoting Zhan, Dongming Ma

**Affiliations:** 1 Ministry of Education Key Laboratory of Chinese Medicinal Resource from Lingnan, School of Pharmaceutical Sciences, Guangzhou University of Chinese Medicine, Guangzhou 510006, China; ypq18273297865@163.com (P.Y.); 15889819550@163.com (B.H.); zhanrt@gzucm.edu.cn (R.Z.); 2 Research Center of Chinese Herbal Resource Science and Engineering, Guangzhou University of Chinese Medicine, Guangzhou 510006, China; 3 Dongguan Institute, Guangzhou University of Chinese Medicine, Dongguan 523808, China; 4 Chinese Medicine Guangdong Laboratory, Hengqin, Zhuhai 519031, China

**Keywords:** *Saussurea involucrata*, glycosyltransferases, rutin biosynthesis, quercetin, isoquercetin, enzyme kinetics, flavonoid glycosylation

## Abstract

*Saussurea involucrata* is a medicinal plant rich in flavonoids, but the glycosyltransferases (GTs) responsible for flavonoid glycosylation in this species remain largely uncharacterized. Here, five GT genes (*SiUGT03*, *SiUGT09*, *SiUGT60*, *SiRhaT68-1*, and *SiRhaT68-2*) were identified. Four of the five proteins (SiUGT03, SiUGT09, SiUGT60, and SiRhaT68-1) possessed an intact PSPG-box, and phylogenetic analysis placed SiUGT03 in the 3GT clade and SiRhaT68-1 in the 1,6GT clade. Recombinant proteins were expressed in *Escherichia coli* as soluble maltose-binding protein (MBP) fusions and purified. In vitro enzymatic assays showed that SiUGT03 catalyzed the 3-O-glucosylation of quercetin to isoquercetin (quercetin-3-O-glucoside), whereas SiRhaT68-1 catalyzed the 1,6-rhamnosylation of isoquercetin to rutin. Among the substrates tested, SiUGT03 was active only toward the flavonols quercetin and kaempferol, while SiRhaT68-1 was active toward isoquercetin and one of the four tested flavone 7-O-glucosides, diosmetin-7-O-glucoside. Kinetic analysis revealed an apparent difference in catalytic efficiency between the two enzymes under the conditions tested (SiUGT03: *K*m = 36.08 μM, *k*cat/*K*m = 0.01983 μM^−1^·s^−1^; SiRhaT68-1: *K*m = 184.70 μM, *k*cat/*K*m = 0.0005755 μM^−1^·s^−1^); however, since the *K*m of SiRhaT68-1 was close to the highest substrate concentration tested, this difference should be regarded as preliminary. These results, obtained from separate in vitro assays, demonstrated that the two enzymes catalyze two consecutive terminal glycosylation steps of rutin biosynthesis and represent promising candidates for further exploration of rutin biosynthesis in *S. involucrata*.

## 1. Introduction

*Saussurea involucrata* (Asteraceae) is a rare medicinal plant native to the high-altitude regions of Xinjiang, China, and is widely used in traditional medicine owing to its anti-inflammatory, antioxidant, and immunomodulatory activities [1]. These activities are largely attributed to its abundant flavonoids, including rutin, quercetin, and kaempferol derivatives [2,3]. Flavonoid glycosylation is a key structural modification that enhances the solubility, stability, and biological activity of these compounds and is catalyzed by UDP-glycosyltransferases (UGTs), which transfer sugar moieties from UDP-sugars to acceptor molecules [4]. Nevertheless, the molecular basis underlying bioactive flavonoid production in *S. involucrata* remains incompletely understood, and the biosynthetic mechanisms underlying flavonoid glycosylation in this species have not been systematically characterized.

Flavonoids are polyphenolic compounds that play critical roles in stress responses and tissue pigmentation, and their accumulation often exhibits organ-specific patterns reflecting distinct biosynthesis and storage strategies [5,6]. Beyond these physiological roles, flavonoid glycosides are of considerable pharmaceutical and industrial interest, because glycosylation improves aglycone bioavailability and pharmacological potency [7]. Although the chemical composition of *S. involucrata* has been extensively studied, the enzymes responsible for the formation of its characteristic flavonoid glycosides, particularly rutin, remain largely unidentified.

Plant UGTs constitute a large multigene family characterized by a conserved C-terminal PSPG-box (plant secondary product glycosyltransferase box) that binds the UDP-sugar donor, together with a variable N-terminal domain that determines acceptor specificity [8]. On the basis of regioselectivity and sugar-donor specificity, UGTs involved in flavonoid biosynthesis are classified into several subfamilies, including 3GT, 5GT, 7GT, 1,2GT, and 1,6GT [9]. Within this classification, 3-O-glucosyltransferases (3GTs) and 1,6-rhamnosyltransferases (1,6RhaTs) catalyze the two consecutive steps of rutin biosynthesis, namely the 3-O-glucosylation of quercetin to isoquercetin and the subsequent 1,6-rhamnosylation of isoquercetin to rutin [10]. In several plant species, including *Fagopyrum esculentum* [11], *Citrus* spp. [12], and *Sophora japonica* [13], UGTs catalyzing these two steps have been functionally characterized and shown to exhibit distinct substrate preferences and catalytic properties. In *S. involucrata*, a UGT (SiUGT72BZ2) has been functionally characterized in the context of syringin biosynthesis [14]. However, the UGTs responsible for rutin biosynthesis in this species have not been identified.

To fill these research gaps, the present study combined gene cloning, recombinant protein expression, in vitro enzymatic assays, enzyme kinetics, and phylogenetic analysis. Specifically, this work aimed to identify and clone the UGT genes potentially involved in flavonoid glycosylation in *S. involucrata*, to characterize the sequence conservation and evolutionary relationships of the encoded proteins, to characterize the catalytic functions of the recombinant enzymes against selected flavonoid substrates, to compare the apparent kinetic parameters of the two enzymes, and to evaluate their substrate preferences and catalytic efficiency in vitro.

This study provides a functional characterization of UGTs involved in rutin biosynthesis in *S. involucrata*, identifying SiUGT03 and SiRhaT68-1 as the enzymes that catalyze the 3-O-glucosylation of quercetin to isoquercetin and the 1,6-rhamnosylation of isoquercetin to rutin, respectively, in vitro. This extended previous functional work on *S. involucrata* UGTs, such as SiUGT72BZ2, by defining two enzymes acting on the terminal steps of rutin biosynthesis. Kinetic analysis suggested an apparent difference in catalytic efficiency between the two steps under the conditions tested, with 3-O-glucosylation appearing more efficient than 1,6-rhamnosylation; however, because the *K*m of SiRhaT68-1 was close to the highest substrate concentration tested, the apparent difference in *k*cat/*K*m should be interpreted with caution, and whether 1,6-rhamnosylation limits flux in planta remains hypothetical and requires further experimental validation. SiUGT03 and SiRhaT68-1 are proposed as candidate enzymes for further investigation of rutin biosynthesis and related flavonoid glycosylation pathways, providing a basis for further engineering studies in microbial or plant systems [15,16].

## 2. Results

### 2.1. Prediction of the Putative Rutin Biosynthetic Pathway and Phylogenetic Analysis

**Figure 1:**
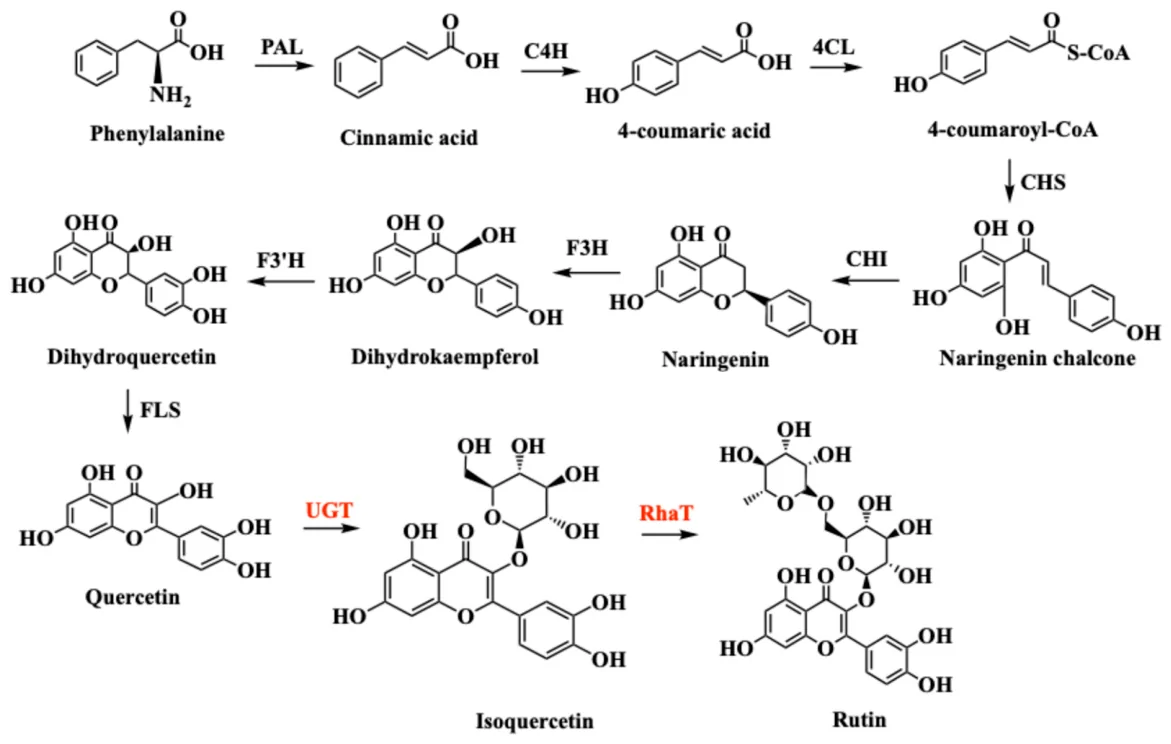
Putative biosynthetic pathway of rutin in *S. involucrata*. PAL, phenylalanine ammonia-lyase; C4H, cinnamate 4-hydroxylase; 4CL, 4-coumarate-CoA ligase; CHS, chalcone synthase; CHI, chalcone isomerase; F3H, flavanone 3-hydroxylase; F3′H, flavonoid 3′-hydroxylase; FLS, flavonol synthase; UGT, UDP-glycosyltransferase; RhaT, rhamnosyltransferase. UGT and RhaT are the key enzymes investigated in this study.

A putative biosynthetic pathway for rutin in *S. involucrata* was proposed (Figure 1). Starting from phenylalanine, the upstream phenylpropanoid pathway is sequentially catalyzed by phenylalanine ammonia-lyase (PAL), cinnamate 4-hydroxylase (C4H), and 4-coumarate-CoA ligase (4CL), which convert phenylalanine into 4-coumaroyl-CoA. Subsequently, chalcone synthase (CHS) and chalcone isomerase (CHI) transform 4-coumaroyl-CoA into naringenin [17,18]. Naringenin is further hydroxylated by flavanone 3-hydroxylase (F3H) and flavonoid 3′-hydroxylase (F3′H) to yield dihydroquercetin, which is then converted to quercetin under the catalysis of flavonol synthase (FLS).

The two terminal glycosylation steps, which represent the core focus of this study, are mediated by UDP-glycosyltransferases (UGTs) and rhamnosyltransferases (RhaTs). UGTs catalyze the glycosylation of quercetin to form isoquercetin, and RhaTs further transfer a rhamnose moiety to isoquercetin, finally generating rutin [19,20]. This pathway integrates upstream phenylpropanoid metabolism and the two terminal glycosylation steps characterized in vitro in this study.

### 2.2. Multiple Sequence Alignment and Phylogenetic Analysis

**Figure 2:**
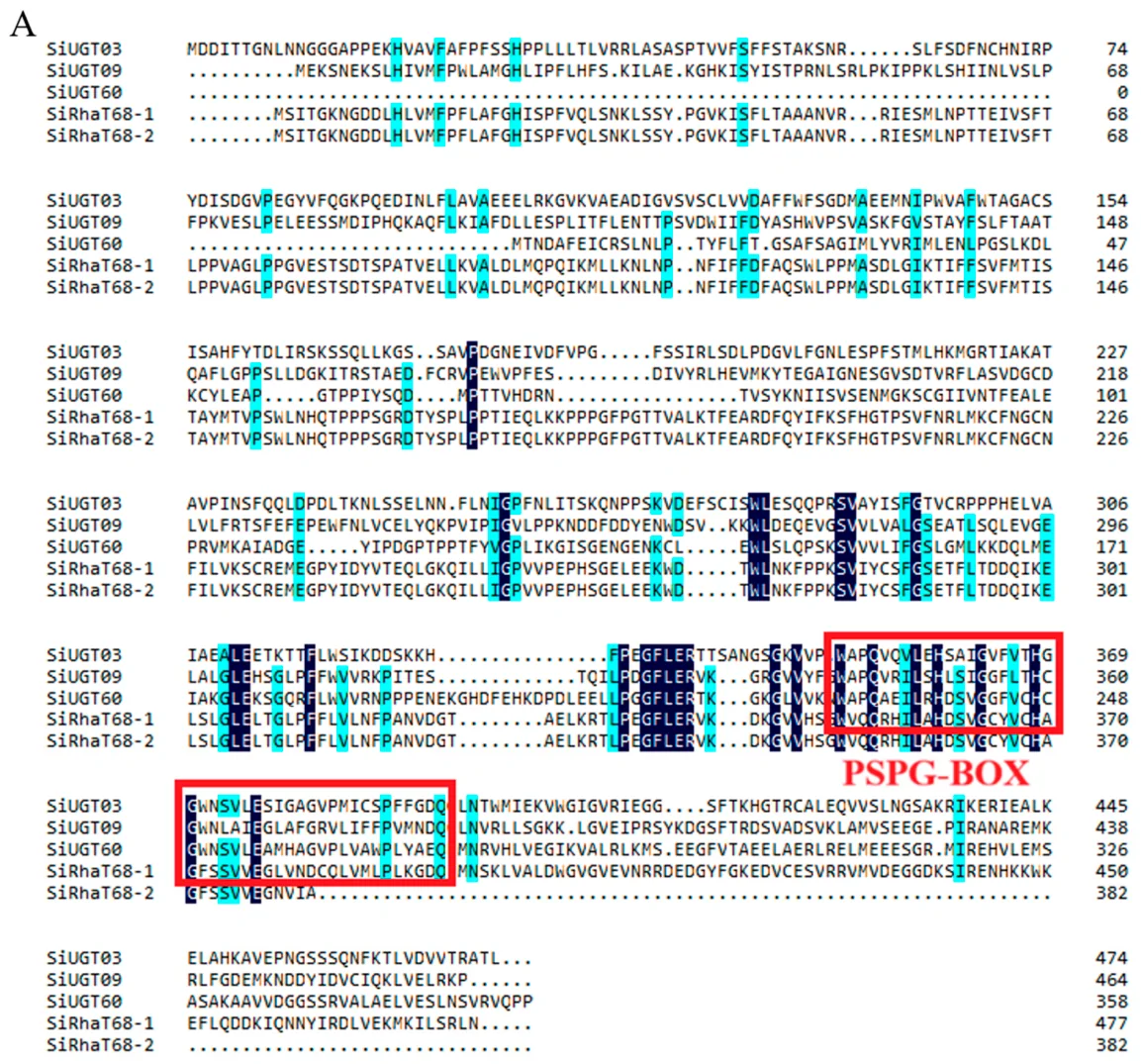

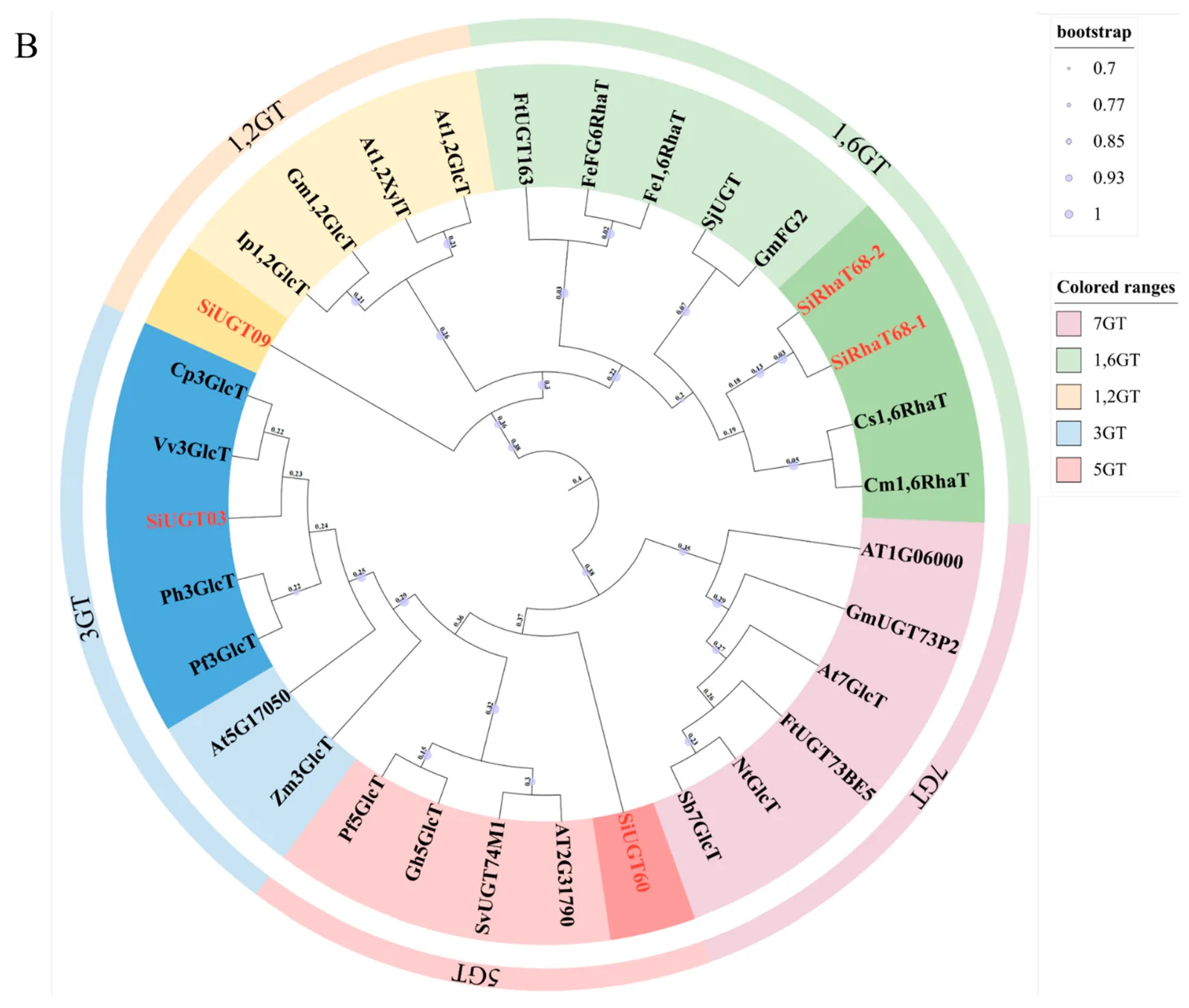
Multiple sequence alignment and phylogenetic tree analysis of SiGTs. (**A**) Conserved amino acid residues are shaded in dark blue (100% identity) and light blue (≥75% identity). The red box represents the conserved PSPG-box domain. (**B**) Phylogenetic tree of SiGTs together with functionally characterized UGT proteins from multiple plants. Colored ranges indicate different glycosyltransferase subfamilies: 7GT (pink), 1,6GT (light green), 1,2GT (yellow), 3GT (blue), and 5GT (light pink). SiGTs are highlighted in red. The scale bar represents substitutions per site.

To investigate the structural conservation and evolutionary relationships of the SiGTs, the deduced amino acid sequences were aligned with several functionally characterized UGTs from other plants (Figure 2A). Four of the five sequences (SiUGT03, SiUGT09, SiUGT60, and SiRhaT68-1) contained an intact PSPG-box (Figure 2A, red box), the core domain responsible for UDP-sugar binding in plant UGTs. Consistent with this sequence alignment, the conserved HCGWNS motif was present in these four proteins with intact PSPG-box, while SiRhaT68-2 lacked a substantial portion of the PSPG-box region and did not harbor the HCGWNS motif. This incomplete PSPG-box in SiRhaT68-2 was consistent with its lack of detectable activity against the tested substrates; whether this represents a natural transcript variant, a truncated cloning product, or a sequence reporting error remained to be determined. In contrast, the N-terminal region was more variable across all SiGTs, which likely contributed to differences in substrate recognition and catalytic specificity. Notably, SiRhaT68-1 exhibited the highest conservation within the PSPG-box and downstream regions, suggesting potential 1,6-rhamnosyltransferase activity. SiUGT03, SiUGT09, and SiUGT60 exhibited greater sequence variation upstream of the PSPG-box, which may be associated with their distinct substrate specificities.

To further clarify the evolutionary position of the SiGTs, a neighbor-joining (NJ) phylogenetic tree was constructed using MEGA 11 with 1000 bootstrap replicates (Figure 2B). The full-length amino acid sequences used for tree construction are provided in Table S1. The tree resolved into distinct clades corresponding to 3GT, 5GT, 7GT, 1,2GT, and 1,6GT. SiUGT03 clustered into the 3GT clade together with characterized 3-O-glycosyltransferases from *Citrus* and *Vitis*, suggesting that SiUGT03 may possess 3-O-glycosyltransferase activity. SiRhaT68-1 clustered into the 1,6GT clade together with characterized 1,6-rhamnosyltransferases involved in rutin biosynthesis (Cs1,6RhaT, Cm1,6RhaT, and Fe1,6RhaT). Given that members of this clade catalyze the glycosylation of flavonol 3-O-glucosides, SiRhaT68-1 is predicted to possess 1,6-rhamnosyltransferase activity and to participate in the final step of rutin biosynthesis. In contrast, SiUGT60 and SiUGT09 were assigned to the 5GT and 1,2GT clades, respectively, suggesting that they may catalyze different glycosylation reactions.

### 2.3. Protein Expression and Purification

**Figure 3:**
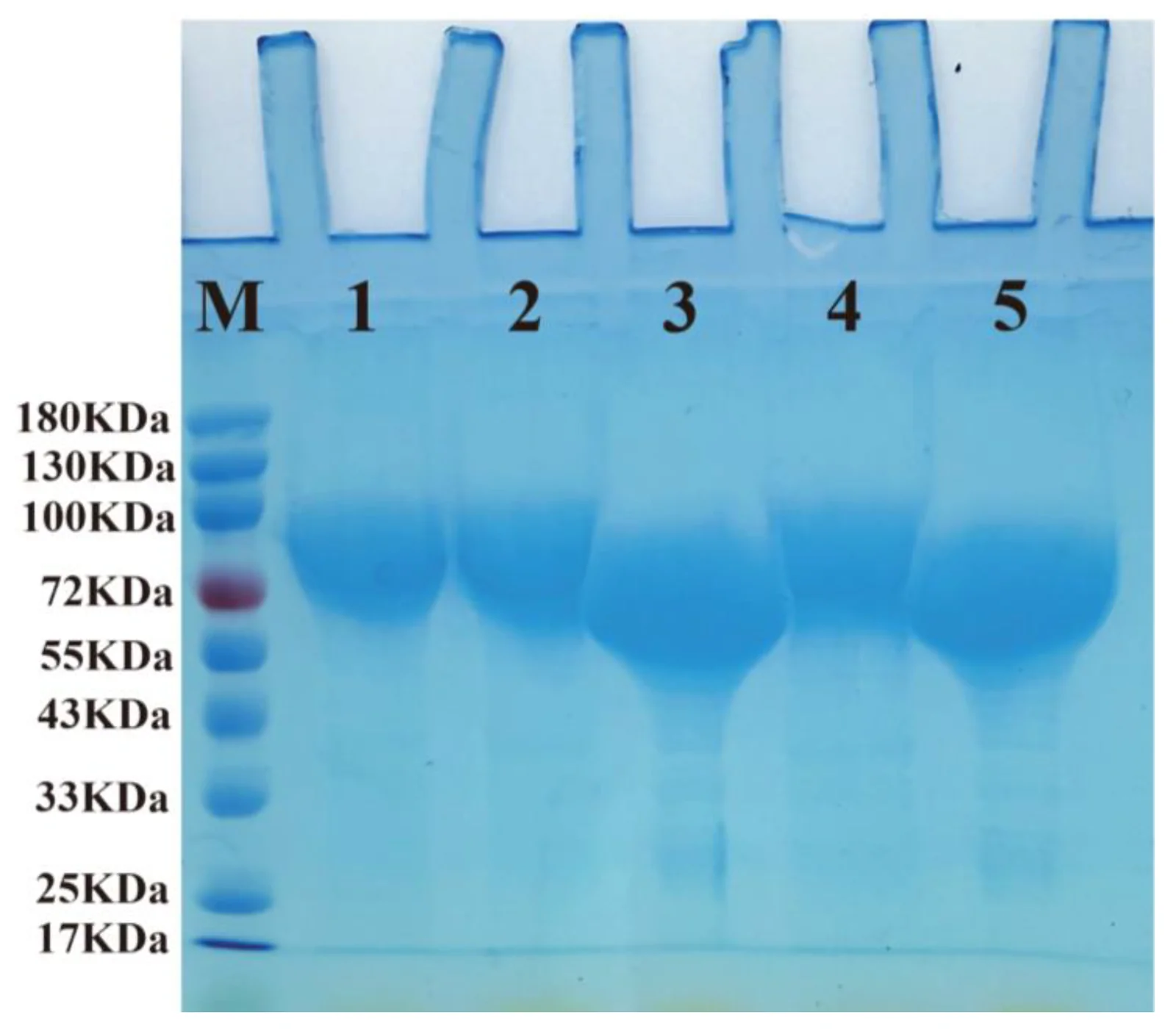
SDS-PAGE analysis of recombinant SiGT proteins. M: Marker; 1: Purified MBP-SiUGT03; 2: Purified MBP-SiUGT09; 3: Purified MBP-SiUGT60; 4: Purified MBP-SiRhaT68-1; 5: Purified MBP-SiRhaT68-2.

SDS-PAGE analysis of the purified proteins revealed specific bands at approximately 82–96 kDa, consistent with the theoretical molecular weights of the MBP-fused SiGTs (Figure 3). The proteins were efficiently expressed in soluble form in the supernatant and were almost completely bound to the MBP affinity column, and high-purity products were obtained in the elution fractions. The final protein concentrations exceeded 1.8 mg/mL, as determined by the bicinchoninic acid (BCA) method, which was sufficient for the subsequent experiments.

### 2.4. In Vitro Enzymatic Reactions

**Figure 4:**
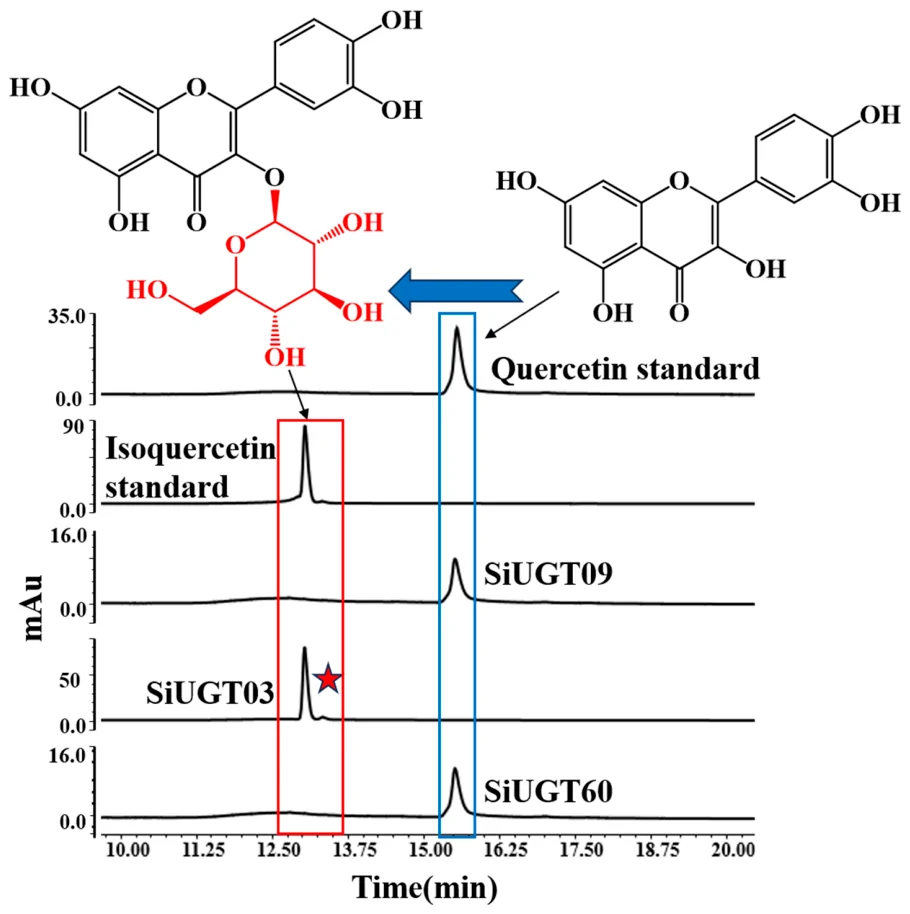
UPLC profiles of in vitro glucosylation reactions of SiUGTs. The blue arrow illustrates the glucosylation reaction from quercetin to isoquercetin. The blue box marks the retention time of the quercetin standard, and the red box marks the retention time of the isoquercetin standard. The red asterisk indicates the target glycosylated product peak catalyzed by SiUGT03.

**Figure 5:**
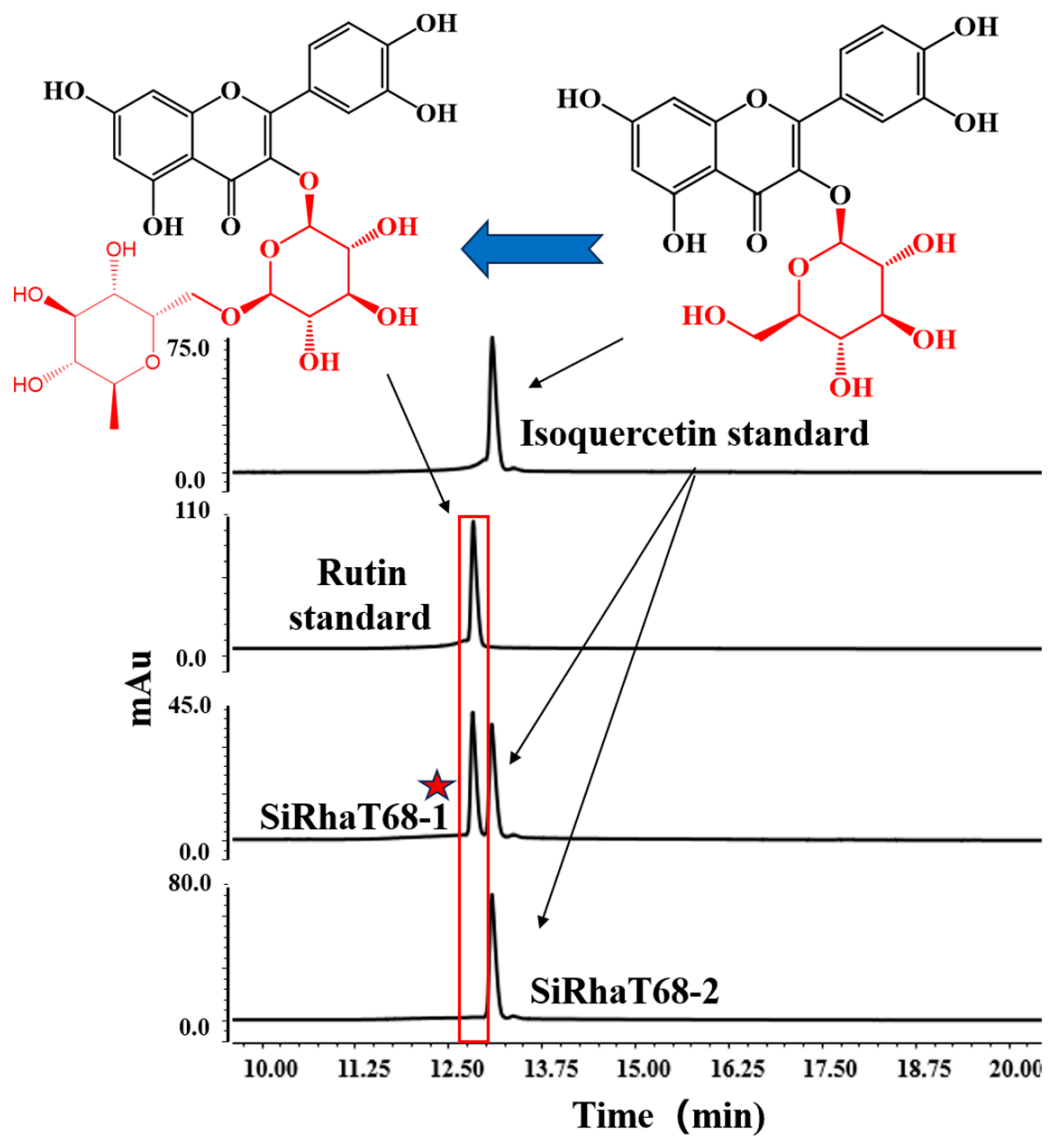
UPLC profiles of in vitro 1,6-rhamnosylation reactions of SiRhaTs. The blue arrow illustrates the 1,6-rhamnosylation reaction from isoquercetin to rutin. The red box marks the retention time of the rutin standard. The red asterisk indicates the target rhamnosylated product peak catalyzed by SiRhaT68-1.

In vitro enzyme assays were performed using quercetin and isoquercetin, the candidate substrates involved in rutin biosynthesis in *S. involucrata*. For reactions using UDP-glucose as the sugar donor, UPLC (ultra-high-performance liquid chromatography) profiles showed substrate peaks for all enzymes, and a product peak appeared in the SiUGT03 reaction. The retention time of this product matched well with that of the isoquercetin standard. Furthermore, LC-MS/MS analysis showed that the quasi-molecular ion peak of the product was *m*/*z* 463.09 [M − H]^−^, consistent with the molecular weight of isoquercetin. These results indicate that SiUGT03 catalyzes the 3-O-glucosylation of quercetin to produce isoquercetin (Figure 4 and Figure S1). In the reaction system with UDP-rhamnose as the sugar donor, UPLC profiles showed substrate peaks, and a product peak appeared in the SiRhaT68-1 reaction. The retention time of this product was consistent with that of the rutin standard. LC-MS/MS analysis showed that the quasi-molecular ion peak was *m*/*z* 609.14 [M − H]^−^, consistent with the molecular weight of rutin. These results indicated that SiRhaT68-1 catalyzes the 1,6-rhamnosylation of isoquercetin to produce rutin (Figure 5 and Figure S1).

### 2.5. Substrate Promiscuity

In the UDP-glucose-dependent reaction system, the SiUGTs were incubated with nine flavonoid compounds, including the flavones apigenin (Figure S2), acacetin (Figure S3), luteolin (Figure S4), diosmetin (Figure S5), hispidulin (Figure S6), and scutellarein (Figure S7), the flavanone eriodictyol (Figure S8), the isoflavone genistein (Figure S9), and the flavonol kaempferol (Figure S10). In addition, quercetin was tested separately in Section 2.4, and SiUGT03 was active toward it. UPLC profiles showed substrate peaks for all reactions. Among the nine compounds tested here, SiUGT03 produced a product peak only with kaempferol as the substrate, and the product retention time matched well with that of the kaempferol-3-O-glucoside standard (Figure S10; Table 1). Together with the results in Section 2.4, SiUGT03 was active toward quercetin and kaempferol, but not toward the other eight compounds tested. In the reaction system with UDP-rhamnose as the sugar donor, the SiRhaTs were incubated with four flavonoid compounds, including the flavone 7-O-glucosides apigenin-7-O-glucoside (Figure S11), acacetin-7-O-glucoside (Figure S12), luteolin-7-O-glucoside (Figure S13), and diosmetin-7-O-glucoside (Figure S14). Substrate peaks were observed in all UPLC profiles, and SiRhaT68-1 produced a product peak only with diosmetin-7-O-glucoside as the substrate (Figure S14; Table 2). In addition, isoquercetin was tested separately in Section 2.4, and SiRhaT68-1 was active toward it. These results indicated that the SiGTs tested here have a narrow substrate range. Among the compounds tested, SiUGT03 acted on the flavonols quercetin and kaempferol, whereas SiRhaT68-1 acted on isoquercetin and one of the four flavone 7-O-glucosides tested, diosmetin-7-O-glucoside. The full substrate spectra of these enzymes remain to be determined.

### 2.6. Enzyme Kinetics of SiUGT03 and SiRhaT68-1

**Figure 6:**
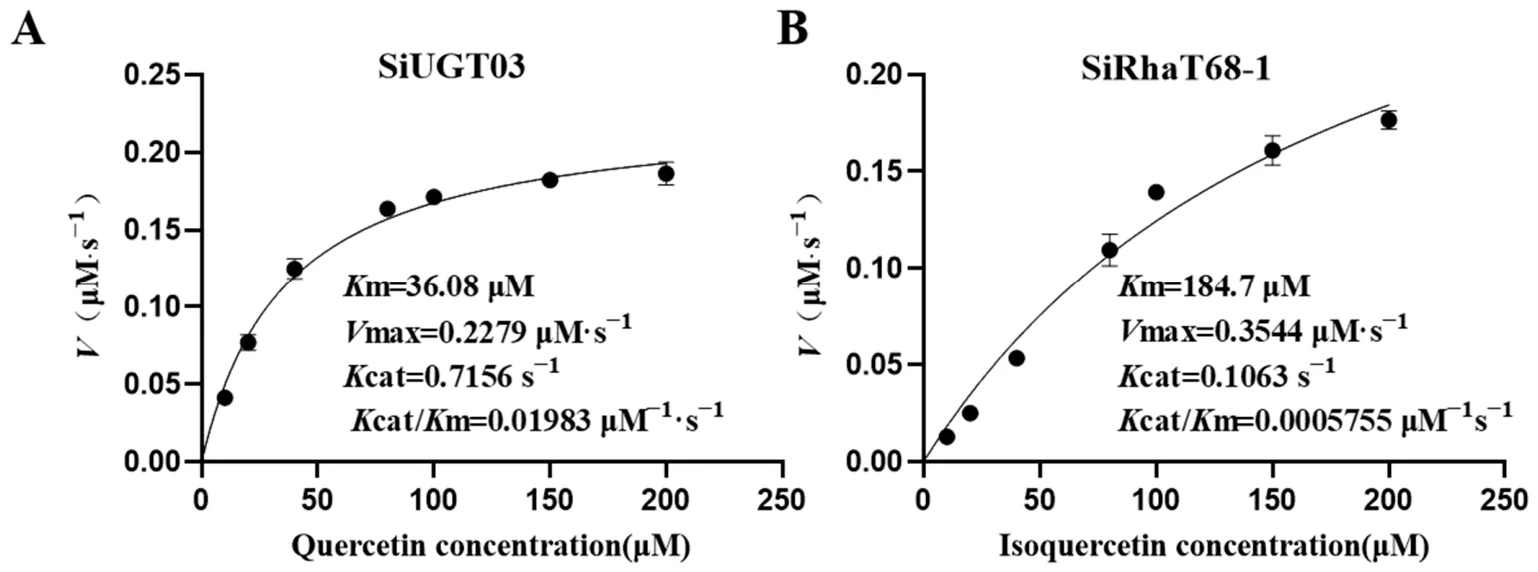
Enzyme kinetics of SiUGT03 and SiRhaT68-1. (**A**) Kinetic curve of SiUGT03 with quercetin as the substrate. (**B**) Kinetic curve of SiRhaT68-1 with isoquercetin as the substrate. *V*max, maximum reaction rate (μM·s^−1^); *K*m, Michaelis constant (μM); kcat, turnover number (s^−1^); *k*cat/*K*m, catalytic efficiency (μM^−1^·s^−1^). Data are shown as mean ± SD (*n* = 3). Initial rates were measured within the linear range of product formation, with substrate consumption <10% of the initial substrate concentration. Solid lines represent non-linear least-squares fitting to the Michaelis–Menten equation. The 95% confidence intervals of fitted kinetic parameters are listed in Table 3. For SiRhaT68-1, the tested maximum substrate concentration was only slightly higher than its fitted *K*m value, leading to relatively large uncertainty in this kinetic parameter.

To further characterize the kinetic properties of SiUGT03 and SiRhaT68-1, enzyme kinetics were analyzed using quercetin and isoquercetin as substrates, respectively (Figure 6). All kinetic curves fitted the Michaelis–Menten equation well (R^2^ > 0.995). The kinetic parameters are summarized in Table 3. SiUGT03 had a *K*m of 36.08 μM (95% confidence interval (CI): 31.00–42.01), indicating a high affinity for quercetin. Its *V*max was 0.2279 μM·s^−1^, its *k*cat was 0.7156 s^−1^, and its *k*cat/*K*m was 0.01983 μM^−1^·s^−1^. These results suggest that SiUGT03 was an efficient flavonol 3-O-glucosyltransferase capable of effectively catalyzing the conversion of quercetin to isoquercetin.

SiRhaT68-1 showed a lower affinity for isoquercetin than SiUGT03 did for quercetin. Its *K*m was 184.7 μM (95% CI: 132.9–269.4), approximately five-fold higher than that of SiUGT03, indicating weaker substrate binding. Its *V*max was 0.3544 μM·s^−1^, its *k*cat was 0.1063 s^−1^, and its *k*cat/*K*m was 0.0005755 μM^−1^·s^−1^, indicating an apparent difference in catalytic efficiency relative to SiUGT03. Overall, the catalytic efficiency of SiUGT03 was higher than that of SiRhaT68-1 under the conditions tested; however, because the *K*m of SiRhaT68-1 was close to the highest substrate concentration tested, this difference should be interpreted with caution. In addition, the *k*cat/*K*m value of SiRhaT68-1 was comparable to previously reported rhamnosyltransferases such as *Fagopyrum esculentum* FeF3G6″RhaT, further supporting its functional assignment as a rhamnosyltransferase in rutin biosynthesis.

Initial reaction rates were confirmed to fall within the linear range of product formation via time-course assays (Figure S15). Linear regression analysis indicated that product formation was linearly proportional to reaction time under the adopted assay conditions, with substrate consumption kept below 10% of the initial substrate concentration.

## 3. Discussion

A central finding of the present study is that SiUGT03 and SiRhaT68-1 catalyze the 3-O-glucosylation of quercetin and the 1,6-rhamnosylation of isoquercetin, respectively, representing two independently demonstrated consecutive reactions of rutin biosynthesis in vitro. This division of catalytic roles resembles the pattern reported for *Fagopyrum esculentum*, *Citrus*, and *Prunus persica* [21], suggesting that rutin-type flavonoid glycoside formation may rely on a relatively conserved enzymatic strategy across different plant lineages. However, because all evidence in this study originates from in vitro assays with recombinant enzymes, whether SiUGT03 and SiRhaT68-1 function in this sequential manner in planta cannot be established from the present data alone. A complete coupled two-enzyme assay together with in planta genetic validation is still required to fully confirm the sequential catalytic capacity of these two enzymes, which would include in planta co-expression analysis and metabolite accumulation verification.

Four of the five SiGT proteins (SiUGT03, SiUGT09, SiUGT60, and SiRhaT68-1) contain the PSPG-box, consistent with the structural hallmarks of the plant UGT family [22,23]. SiRhaT68-2 lacks a complete PSPG-box, which may account for its undetectable catalytic activity. Whether this truncation represents a natural transcript variant, a cloning artifact, or a sequence annotation error remains to be determined. Notably, SiUGT03, SiUGT09, and SiUGT60 display considerable sequence divergence in the region upstream of the PSPG-box, a region generally implicated in acceptor recognition. Whether this sequence divergence corresponds directly to their functional differentiation remains difficult to determine. On the one hand, the substrate spectrum of SiUGT03 is restricted to flavonols quercetin and kaempferol, resembling the behavior of the comparable 3-O-glucosyltransferase from *Fagopyrum esculentum*; on the other hand, SiRhaT68-1 shows activity toward isoquercetin and one of the four tested flavone 7-O-glucosides, diosmetin-7-O-glucoside, indicating that its acceptor promiscuity may extend beyond flavonol 3-O-glucosides but is not yet fully defined. These observations are consistent with the general view that the N-terminal domain governs acceptor breadth whereas the C-terminal region determines sugar-donor recognition [24]; however, since no domain-swapping or site-directed mutagenesis experiments were performed in this study, this interpretation remains inferential rather than verified.

The apparent difference in catalytic efficiency between the two enzymes revealed by kinetic analysis constitutes another observation worthy of discussion. The *K*m of SiUGT03 for quercetin was 36.08 μM, whereas the *K*m of SiRhaT68-1 for isoquercetin was 184.7 μM, approximately five-fold higher; correspondingly, the apparent *k*cat/*K*m of SiUGT03 was higher than that of SiRhaT68-1. This difference may be interpreted in several ways. First, this raises the hypothesis of a division of catalytic roles for flux control between these two biosynthetic steps in vivo, and whether the 1,6-rhamnosylation step limits metabolic flux in planta remains to be verified. Second, the lower substrate affinity of SiRhaT68-1 may also be related to the selective pressure to discriminate between glycosylated and non-glycosylated flavonoid acceptors, such that selectivity is achieved at the expense of turnover efficiency. Third, because the kinetic measurements employed purified recombinant proteins and a single substrate, factors such as in vivo substrate concentration, enzyme abundance, and compartmentalization were not taken into account; consequently, the above interpretations should be regarded as possibilities rather than conclusions. Importantly, because the *K*m of SiRhaT68-1 was close to the highest substrate concentration tested, the kinetic parameters for this enzyme, and therefore the apparent difference in catalytic efficiency, should be considered preliminary. In addition, the catalytic efficiency of SiRhaT68-1 was of the same order of magnitude as that of *Fagopyrum esculentum* FeF3G6″RhaT, which lends some support to its functional assignment while also suggesting that characterized 1,6-rhamnosyltransferases can exhibit relatively low turnover capacity. Structural studies of *Arabidopsis* UGT89C1 have proposed that donor-specific recognition of UDP-rhamnose by rhamnosyltransferases constrains the active-site architecture [25], which may offer a structural explanation for this phenomenon; however, whether this hypothesis applies to SiRhaT68-1 still requires verification through crystal structures or mutagenesis experiments.

From a metabolic engineering perspective, the apparent difference in catalytic efficiency between the two enzymes suggests that the rhamnosylation step may merit attention when the rutin pathway is reconstituted in heterologous hosts. This echoes previous reports describing reconstitution of quercetin O-diglycoside biosynthesis in *Escherichia coli*, in which the two glycosylation activities had to be maintained at an appropriate ratio to support efficient accumulation. Nevertheless, no heterologous pathway assembly experiments were conducted in this study, and this inference should therefore be regarded only as a guide for subsequent work.

Phylogenetic analysis assigned SiUGT03 to the 3GT clade and SiRhaT68-1 to the 1,6GT clade, and the phylogenetic positions of both are broadly consistent with the in vitro enzymatic results. This consistency lends some additional credibility to the functional assignments. However, phylogenetic position alone cannot directly demonstrate in vivo function, particularly since SiRhaT68-2, although highly homologous to SiRhaT68-1, lacks a complete PSPG-box and produces no detectable product under the reaction conditions employed here; whether it possesses alternative substrate preferences or requires different reaction conditions remains to be examined. Similarly, although SiUGT09 and SiUGT60 were assigned to the 5GT and 1,2GT clades, respectively, neither displays activity toward the substrates tested, implying that their physiological substrates may lie outside the compound set selected here. These unresolved issues highlight the inherent limitations of inferring UGT function solely from sequence homology and limited substrate screening.

The present study is also subject to several methodological limitations. First, the substrate screening covered only nine acceptors for the glucosyltransferases and four for the rhamnosyltransferases, which is insufficient to define the full substrate spectrum and sugar-donor preferences of the five SiGTs. Second, all enzymatic data were obtained from MBP fusion proteins expressed in *E. coli*, and whether the fusion tag affects folding, oligomeric state, or activity has not been systematically evaluated. Third, the expression levels of the individual genes across different tissues or developmental stages were not determined; consequently, whether SiUGT03 and SiRhaT68-1 are preferentially expressed in specific organs such as fruits or leaves and are thereby correlated with rutin accumulation currently lacks direct evidence. Fourth, the kinetic parameters were derived from in vitro measurements using purified proteins, and for SiRhaT68-1 the *K*m was close to the highest substrate concentration tested; these values may therefore differ from the actual catalytic environment in vivo. These limitations imply that the present results should be understood as a preliminary functional assignment of UGTs in *S. involucrata* rather than a complete elucidation of the in vivo pathway.

Nevertheless, this study provides several testable leads for subsequent research on flavonoid glycoside biosynthesis in *S. involucrata*. SiUGT03 and SiRhaT68-1 may serve as candidate genes for further in planta functional validation, heterologous pathway assembly, and structure–function studies. At the same time, the functional gaps remaining for SiUGT09, SiUGT60, and SiRhaT68-2 suggest that additional, as yet unidentified flavonoid glycosylation reactions may exist in this species. Future integration of transcriptomic, metabolomic, and genetic transformation approaches should help to resolve the flavonoid glycoside biosynthetic network of *S. involucrata* more completely and provide a basis for the rational engineering of rutin and related glycosides.

## 4. Materials and Methods

### 4.1. Plant Materials

*Saussurea involucrata* plantlets were provided by Shihezi University (Xinjiang, China) and identified by Prof. Dongming Ma. Tissue-cultured plantlets were maintained at Guangzhou University of Chinese Medicine (Guangzhou, Guangdong, China). Healthy plantlets were flash-frozen in liquid nitrogen and stored at −80 °C in an ultra-low temperature freezer (Haier, Qingdao, Shandong, China) until RNA extraction. Three independent biological replicates were prepared for subsequent RNA extraction.

### 4.2. Bioinformatics Analysis of SiGTs

Transcriptomic datasets used in this study were generated from whole-plant tissues of vigorously growing *S. involucrata* tissue-cultured plantlets, with three independent biological replicates subjected to RNA-seq library construction and sequencing by GrandOmics (Wuhan, Hubei, China). Candidate glycosyltransferase genes were mined via BLASTP searches against the *S. involucrata* transcriptome assembly, using two biochemically characterized glycosyltransferases, *Fagopyrum esculentum* FUGT73BE5 (GenBank: MH197414.1) and *Sophora japonica* UGT78K14 (GenBank: OQ934110.1), as query sequences. Sequences were filtered using the following thresholds of E-value ≤ 1 × 10^−10^ and amino acid identity ≥ 35%, yielding five high-confidence SiGT candidate sequences for subsequent functional analysis.

The deduced amino acid sequences of candidate SiGTs were further subjected to BLASTP against the NCBI non-redundant protein database. Conserved protein domains were predicted using the Pfam database (Pfam 35.0, https://pfam.xfam.org/, accessed 18 October 2026). The MEME suite (MEME 5.5.5, https://meme-suite.org/, accessed 18 October 2026) was utilized to characterize the distribution of functional motifs, aiding assessment of whether these candidates belonged to the glycosyltransferase family.

Amino acid sequences of experimentally validated glycosyltransferases with characterized catalytic sites were retrieved from the NCBI protein database. The sequences of the target genes cloned in this study were aligned with these reference sequences using DNAMAN (https://www.lynnon.com/, accessed 20 October 2026). A phylogenetic tree was constructed in MEGA 11.0.13 using the neighbor-joining method with 1000 bootstrap replicates [26]. This analysis allowed preliminary assignment of the target genes to glycosyltransferase subfamilies, providing an evolutionary basis for subsequent functional validation.

### 4.3. Gene Cloning of SiGTs

Healthy *S. involucrata* plantlets were flash-frozen in liquid nitrogen and fully ground into fine powder. Total RNA was extracted from approximately 0.1 g of powder using a plant total RNA extraction kit (Aikerui Biotechnology Co., Ltd., Changsha, China). RNA integrity was verified by agarose gel electrophoresis, and RNA purity was assessed using an ultra-micro UV spectrophotometer (Eppendorf Life Sciences, Hamburg, Germany). First-strand cDNA was synthesized with the FastKing cDNA First-Strand Synthesis Kit (Tiangen Biotech, Beijing, China), using total RNA as template. Specific primers were designed on the basis of the CDS sequences of the five target genes (Table S2) and synthesized by Guangzhou Tsingke Biotechnology Co., Ltd. (Guangzhou, China). PCR amplification was performed in a 25 μL reaction containing 12.5 μL PrimeSTAR Max Premix (Takara Bio, Kusatsu, Shiga, Japan), 1 μL each of the upstream and downstream primers, 1 μL cDNA template, and 9.5 μL nuclease-free water (Takara Bio, Kusatsu, Shiga, Japan). The reaction conditions were as follows: initial denaturation at 98 °C for 3 min; 35 cycles of denaturation at 98 °C for 15 s, annealing at (Tm − 5) °C for 30 s, and extension at 72 °C for 45 s; and a final extension at 72 °C for 5 min. Amplification products were separated by 1% agarose gel electrophoresis, and target fragments were gel-purified and ligated into the pLB cloning vector (Tiangen Biotech, Beijing, China). The constructs were transformed into *Escherichia coli* DH5α competent cells (Weidi Biotechnology, Shanghai, China), and positive recombinant plasmids were obtained after ampicillin (Sangon Biotech, Shanghai, China) resistance screening, colony PCR identification, and sequencing validation.

### 4.4. Protein Expression and Purification

Homologous arm primers were designed (Table S2), and the five SiGT fragments were inserted into the pMAL-c5X vector using the In-Fusion cloning method (Takara Bio, Kusatsu, Shiga, Japan) to generate the recombinant expression vectors pMAL-SiGTs. These vectors were transformed into *Escherichia coli* DH5α competent cells, and positive recombinant plasmids were obtained after ampicillin resistance screening, colony PCR, and sequencing validation. The pMAL-SiGT plasmids were then transformed into *Escherichia coli* BL21(DE3) competent cells (Weidi Biotechnology, Shanghai, China). Positive single colonies were inoculated into LB liquid medium containing ampicillin and cultured at 37 °C with shaking at 220 rpm until OD600 reached approximately 0.6. Protein expression was induced by adding IPTG (Isopropyl β-D-1-thiogalactopyranoside) to a final concentration of 0.1 mM, followed by incubation at 16 °C with shaking at 180 rpm for 20 h. Cells were harvested by centrifugation, resuspended in MBP buffer, and disrupted by ultrasonication. The crude enzyme solution was obtained as the supernatant after centrifugation at 12,000 rpm for 20 min. Purification was performed using a Dextrin Beads 6FF affinity chromatography column (Vazyme Biotech, Nanjing, China), with sequential collection of the flow-through, wash, and elution fractions. The eluted product was desalted using a PD-10 desalting column (Cytiva, Uppsala, Sweden). Aliquots (20 μL) of the crude enzyme, flow-through, wash, elution, and desalted fractions were mixed with 5 μL 5× SDS loading buffer, denatured at 95–98 °C for 10 min, and analyzed by SDS-PAGE to assess recombinant protein purity and band distribution. Protein concentration was determined by the BCA method using absorbance measured with a microplate reader (Tecan, Männedorf, Switzerland).

### 4.5. In Vitro Enzymatic Reactions

UDP-glucose and UDP-rhamnose were used as sugar donors. For the glucosyltransferases, nine representative flavonoids were selected as substrates: the flavones apigenin, acacetin, luteolin, diosmetin, scutellarein, and hispidulin; the flavonol quercetin and kaempferol; the flavanone eriodictyol; and the isoflavone genistein (Yuanye Bio-Technology, Shanghai, China). For the rhamnosyltransferases, isoquercetin, apigenin-7-O-glucoside, luteolin-7-O-glucoside, acacetin-7-O-glucoside, and diosmetin-7-O-glucoside (Yuanye Bio-Technology, Shanghai, China) were used as substrates to verify the in vitro catalytic ability of the recombinant SiGT proteins. The formation of new products was determined by comparing the UPLC profiles of the reaction group with those of the inactivated control group, and the corresponding flavonoid glycoside standards were used as references to determine product retention times.

The glucosyltransferase reaction was performed in a final volume of 200 μL containing 50 mM Tris(hydroxymethyl)aminomethane hydrochloride (Tris-HCl, pH 7.4), 2 mM Dithiothreitol (DTT), 2 mM MgCl_2_, 200 μM UDP-glucose, 20 μM flavonoid substrate, and 30 μg purified recombinant protein. Stock solutions of DTT, MgCl_2_, and UDP-glucose were prepared at 100 mM, 100 mM, and 20 mM, respectively, and were added in volumes of 4 μL, 4 μL, and 2 μL. The flavonoid substrate was added from a 2 mM stock solution prepared in methanol to give a final concentration of 20 μM. The reaction was initiated by the addition of the purified enzyme.

The rhamnosyltransferase reaction was performed in a final volume of 100 μL containing 50 mM Tris-HCl (pH 7.4), 200 μM UDP-rhamnose, 20 μM flavonoid substrate, and 30 μg purified recombinant protein. UDP-rhamnose was added from a 20 mM stock solution to give a final concentration of 200 μM, and the flavonoid substrate was added from a 2 mM stock solution to give a final concentration of 20 μM. The reaction was initiated by the addition of the purified enzyme. Both reaction mixtures were incubated at 37 °C for 3 h. Three biological replicates were prepared for each group, and inactivated recombinant protein (100 °C water bath for 10 min) was used as a negative control. Reactions were terminated by adding 300 μL methanol, and the mixture was vortexed for 1 min to denature the enzyme and centrifuged at 12,000 rpm for 10 min to remove protein precipitates. The supernatant was filtered through a 0.22 μm membrane (Jinteng, Tianjin, China) into a liquid chromatography vial (Biosharp, Hefei, China) for UPLC analysis. UPLC analysis was performed with mobile phase A consisting of 0.1% formic acid aqueous solution and mobile phase B consisting of acetonitrile (HPLC-grade, Merck, Darmstadt, Germany). The injection volume was 10 μL, the flow rate was 0.4 mL/min, and the gradient elution program was as follows: 0–3 min, 15% B; 3–5 min, 15–38% B; 5–9 min, 38–60% B; 9–16 min, 60–80% B; 16–21 min, 80–90% B; 21–25 min, 15% B. The detection wavelength was 330 nm, a commonly used detection wavelength for flavonoids.

The prepared samples were analyzed using a Thermo Q Exactive Plus ultra-high-performance liquid chromatography–tandem mass spectrometry (UPLC-MS/MS) system (ThermoFisher Scientific, Waltham, MA, USA) equipped with an Accucore C18 column (100 × 4.6 mm, 2.6 μm, Thermo Fisher Scientific, Waltham, MA, USA). Mobile phase A was 0.1% formic acid (HPLC-grade, Merck, Darmstadt, Germany) aqueous solution, and mobile phase B was acetonitrile. The injection volume was 1 μL, the column temperature was 35 °C, and the flow rate was 0.2 mL/min. The gradient program was as follows: 0–3 min, 15% B; 3–5 min, linear increase to 38% B; 5–9 min, increase to 60% B; 9–16 min, increase to 80% B; 16–21 min, increase to 90% B; 21–22 min, maintain 90% B; 22–23 min, rapidly decrease to 15% B; 23–25 min, maintain 15% B for equilibration. Mass spectrometry analysis was performed with an electrospray ionization (ESI) source. The ion source temperature was set to 550 °C, the ion source voltage was −4500 V, the curtain gas pressure was 35 psi, and the collision-induced dissociation (CID) energy was set to 2 eV. The declustering potential (DP) and collision energy (CE) were optimized for the target compounds.

### 4.6. Enzyme Kinetics Analysis

To compare the catalytic activities of SiUGT03 and SiRhaT68-1, substrate concentration gradients of 10 μM, 20 μM, 40 μM, 80 μM, 100 μM, 150 μM, and 200 μM were used. For SiUGT03 with quercetin as substrate, 6 μg of purified protein was used and the reaction proceeded for 10 min. For SiRhaT68-1 with isoquercetin as substrate, 32 μg of purified protein was used and the reaction proceeded for 30 min. Other conditions were the same as those described in Section 4.5. Reaction initial rates were determined within the linear range of product formation, which was validated by time-course assays and linear regression analysis, with substrate consumption less than 10% of the initial substrate concentration. Three biological replicates were prepared for each group. The molar concentration of each MBP-fused enzyme was calculated by dividing the measured protein mass concentration by the theoretical molecular weight of the recombinant fusion protein. The turnover constant *k*cat was calculated as *V*max divided by the molar concentration of the MBP-fused enzyme. This calculation used the total molar concentration of the purified MBP-fused protein, without correction for the fraction of catalytically active enzyme in the preparation. After UPLC detection, the Michaelis–Menten equation was fitted, and the kinetic parameters *V*max, *K*m, *k*cat, and *k*cat/*K*m were calculated. The 95% confidence intervals (CI) for *V*max and *K*m were determined by nonlinear regression using the Michaelis–Menten model in GraphPad Prism 9.5.1 (GraphPad Software, Boston, MA, USA) with a *K*m > 0 constraint.

## 5. Conclusions

In this study, five glycosyltransferase genes were identified from *S*. *involucrata*, two of which were functionally characterized in vitro. SiUGT03 catalyzed the 3-O-glucosylation of quercetin to isoquercetin, while SiRhaT68-1 catalyzed the 1,6-rhamnosylation of isoquercetin to rutin. Kinetic analysis revealed an apparent difference in catalytic efficiency between the two enzymes under the conditions tested; however, complete substrate saturation for SiRhaT68-1 could not be achieved due to the limited aqueous solubility of isoquercetin. Therefore, the observed difference in catalytic efficiency should be regarded as preliminary. These findings, obtained from separate in vitro assays, provide molecular insights into flavonoid glycosylation in *S. involucrata* and identify SiUGT03 and SiRhaT68-1 as candidate enzymes for further investigation of rutin biosynthesis and related glycosides.

## Figures and Tables

**Table 1:** Substrate specificity of SiUGTs.

Categories	Substrate	Structure	SiUGT03	SiUGT09	SiUGT60
Flavones	Apigenin	** 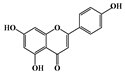 **	n.d.	n.d.	n.d.
	Acacetin	** 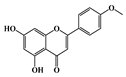 **	n.d.	n.d.	n.d.
	scutellarein	** 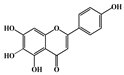 **	n.d.	n.d.	n.d.
	hispidulin	** 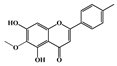 **	n.d.	n.d.	n.d.
	Luteolin	** 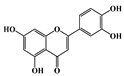 **	n.d.	n.d.	n.d.
	diosmetin	** 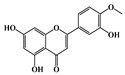 **	n.d.	n.d.	n.d.
Flavanones	eriodictyol	** 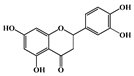 **	n.d.	n.d.	n.d.
Isoflavones	Genistein	** 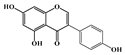 **	n.d.	n.d.	n.d.
Flavonols	kaempferol	** 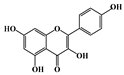 **	**+**	n.d.	n.d.
	quercetin	** 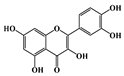 **	**+**	n.d.	n.d.

In vitro enzymatic activity of SiUGTs toward different flavonoid substrates. The reactions were performed using UDP-glucose as the sugar donor. Substrates tested included flavones, flavanones, isoflavones and flavonols. The symbol “**+**” indicates formation of a glycosylated product; “n.d.” (not detected) indicates no detectable product.

**Table 2:** Substrate specificity of SiRhaTs.

Categories	Substrate	Structure	SiRhaT68-1	SiRhaT68-2
Flavone glycosides	apigenin-7-O-glucoside	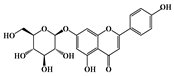	n.d.	n.d.
	acacetin-7-O-glucoside	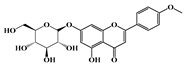	n.d.	n.d.
	luteolin-7-O-glucoside	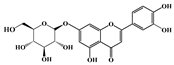	n.d.	n.d.
	diosmetin-7-O-glucoside	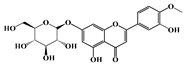	**+**	n.d.
Flavonol glycosides	isoquercetin	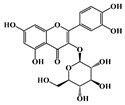	**+**	n.d.

In vitro enzymatic activity of SiRhaTs toward different flavonoid substrates. The reactions were performed using UDP-rhamnose as the sugar donor. Substrates tested included flavone 7-O-glucosides and flavonol 3-O-glucosides. The symbol “**+**” indicates formation of a rhamnosylated product; “n.d.” (not detected) indicates no detectable product.

**Table 3:** Enzyme kinetics analysis. Kinetic parameters of SiUGT03 and SiRhaT68-1.

Enzyme Kinetic Parameters	SiUGT03	SiRhaT68-1
*V*max (μM·s^−1^)	0.2279 (95% CI: 0.2176–0.2392)	0.3544 (95% CI: 0.2947–0.4483)
*K*m (μM)	36.08 (95% CI: 31.00–42.01)	184.7 (95% CI: 132.9–269.4)
*k*cat (s^−1^)	0.7156	0.1063
*k*cat/*K*m (μM^−1^·s−^1^)	0.01983	0.0005755

Note: Data are shown as mean ± SD (*n* = 3). CI: confidence interval.

## Data Availability

The original contributions presented in this study are included in the article/Supplementary Materials. Further inquiries can be directed to the corresponding author. Raw UPLC and kinetic data are available in the manuscript and Supplementary Figures. The gene sequences reported in this study will be deposited in GenBank upon acceptance. Raw transcriptomic reads will be submitted to NCBI SRA after acceptance, with accession numbers provided at publication.

## Supplementary Materials

The following supporting information can be downloaded at https://www.mdpi.com/article/10.3390/ijms27219660/s1.

## Abbreviations

The following abbreviations are used in this manuscript:

LC-MS/MS	liquid chromatography–tandem mass spectrometry
UPLC	ultra-high-performance liquid chromatography
CI	confidence interval
MBP	maltose-binding protein
CDS	coding sequence
ESI	electrospray ionization
UDP-rhamnose	uridine 5′-diphosphate-rhamnose
UDP-glucose	uridine 5′-diphosphate-glucose
NJ	neighbor-joining
SDS-PAGE	sodium dodecyl sulfate–polyacrylamide gel electrophoresis
