# Supplementary material for: Functional Characterization of Glycosyltransferases from Saussurea involucrata Involved in Rutin Biosynthesis
Source: Int J Mol Sci. 2026 Oct 28;27(21):9660. doi: 10.3390/ijms27219660 (PMC13763011; doi:10.3390/ijms27219660)
Supplement: Supplementary file 1 [file ijms-27-09660-s001.zip › Figures S1-S15.pdf]

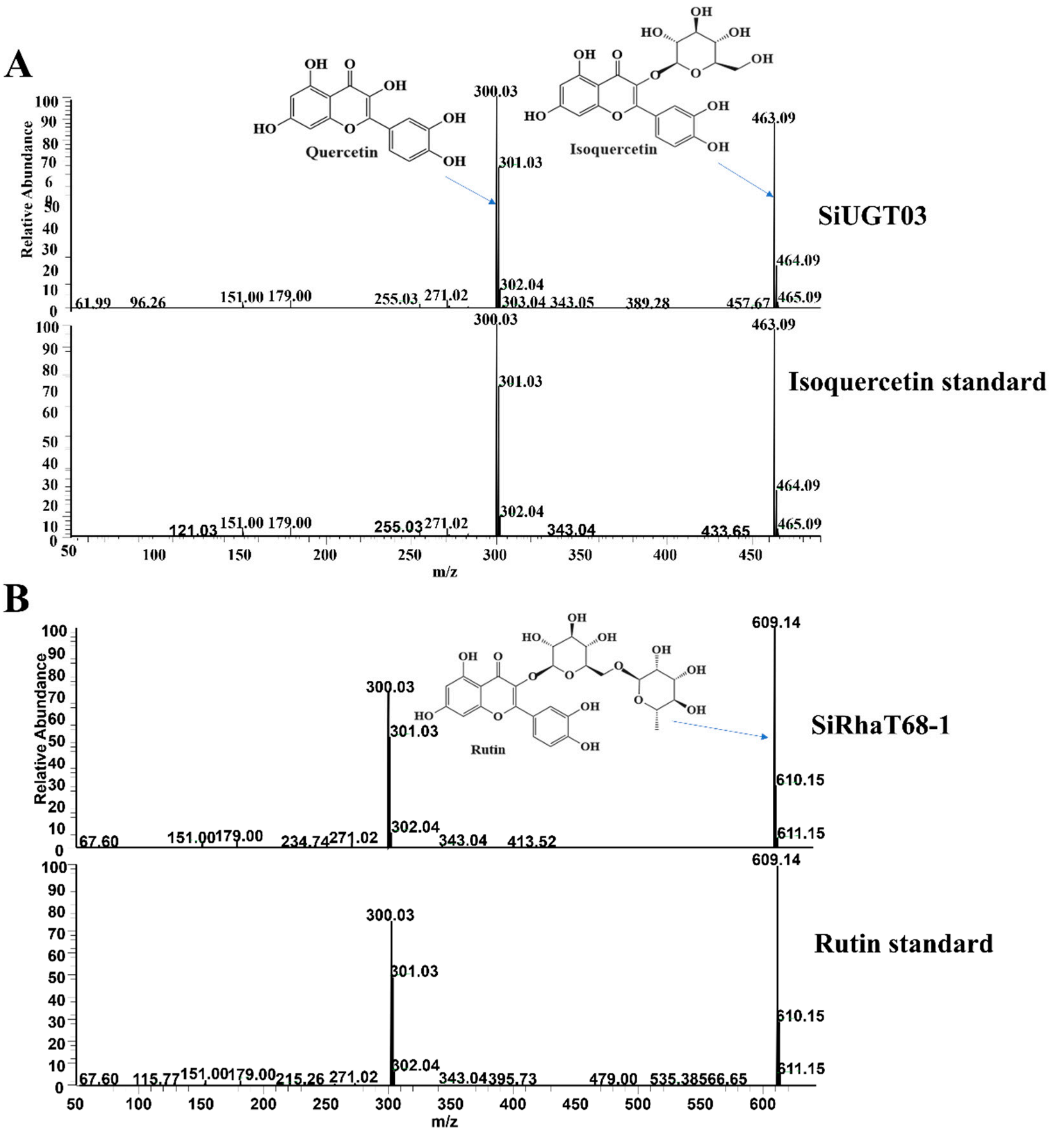

Figure S1. LC-MS/MS results of SiUGT03 and SiRhaT68-1. (A) Mass spectrum of the enzymatic product of SiUGT03 using quercetin as the substrate. The quasi-molecular ion peak at m/z 463.09 [M-H]$^-$ is consistent with that of the isoquercetin standard. (B) Mass spectrum of the enzymatic product of SiRhaT68-1 using isoquercetin as the substrate. The quasi-molecular ion peak at m/z 609.14 [M-H]$^-$ is consistent with that of the rutin standard.

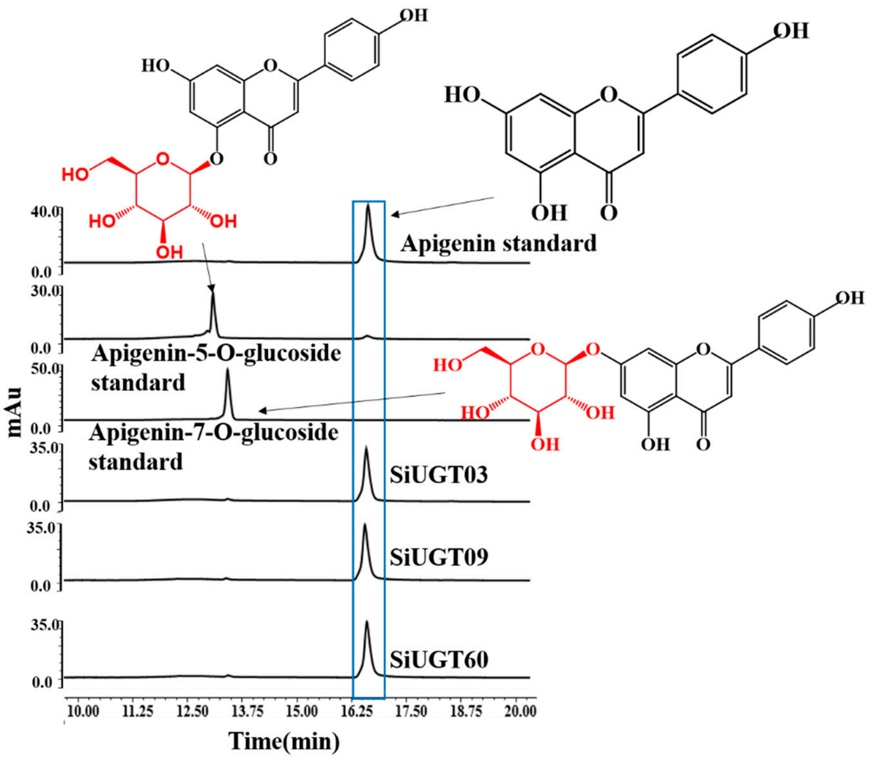

Figure S2. UPLC profiles of in vitro glucosylation reactions of SiUGTs with apigenin substrate.

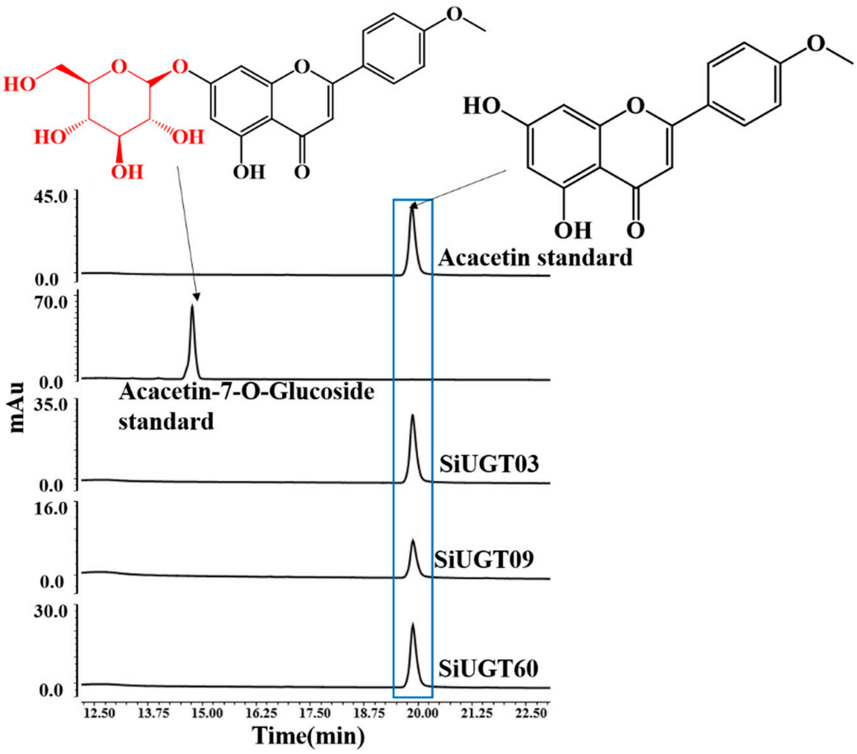

Figure S3. UPLC profiles of in vitro glucosylation reactions of SiUGTs with acacetin substrate.

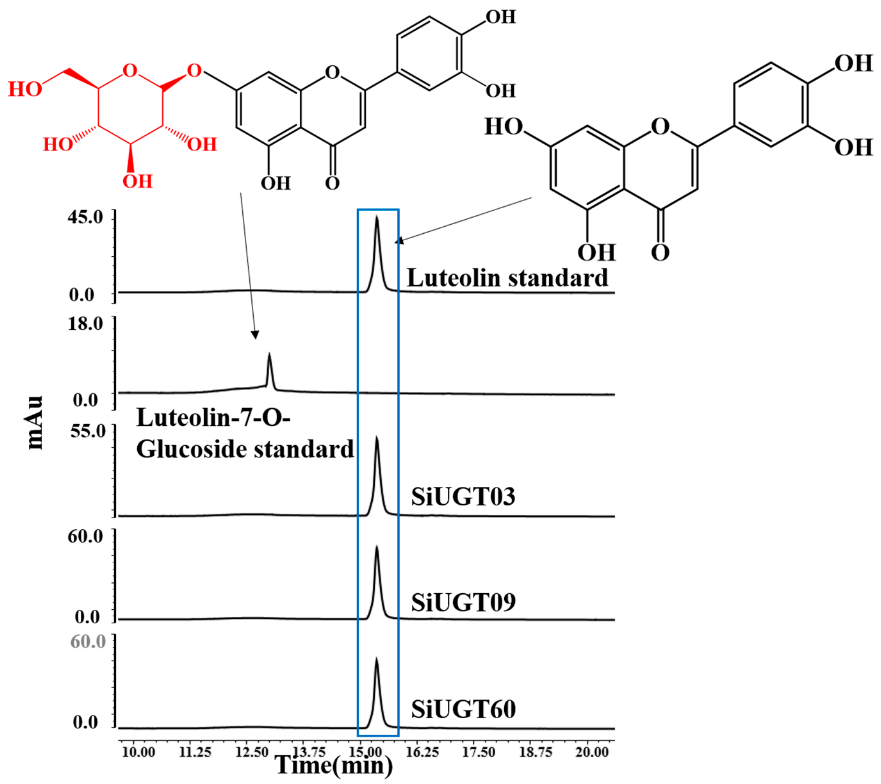

Figure S4. UPLC profiles of in vitro glucosylation reactions of SiUGTs with luteolin substrate.

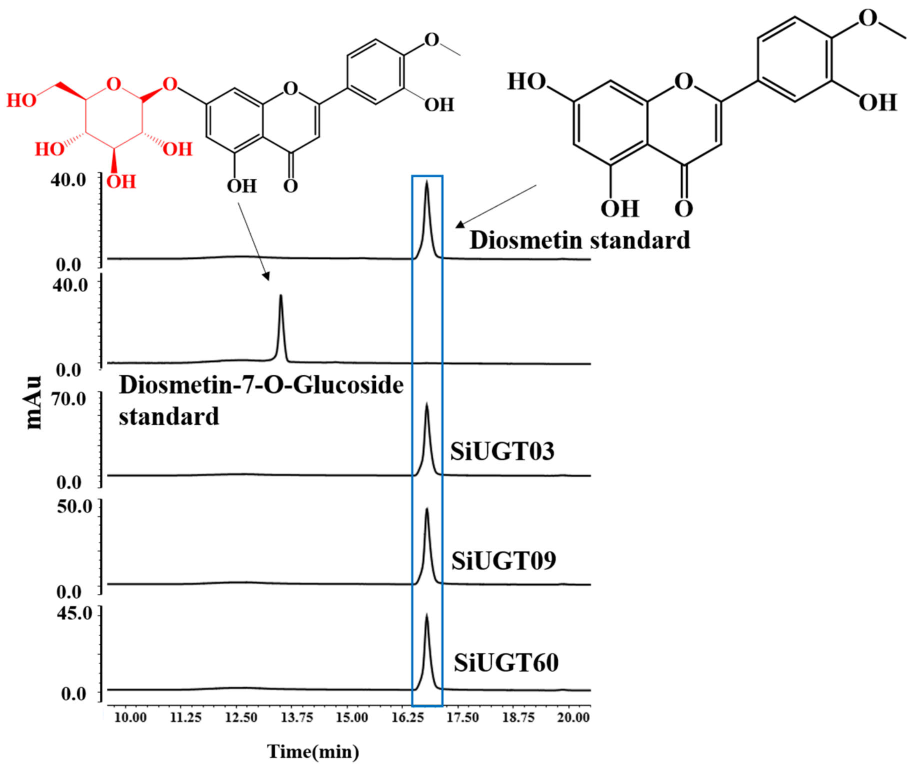

Figure S5. UPLC profiles of in vitro glucosylation reactions of SiUGTs with diosmetin substrate.

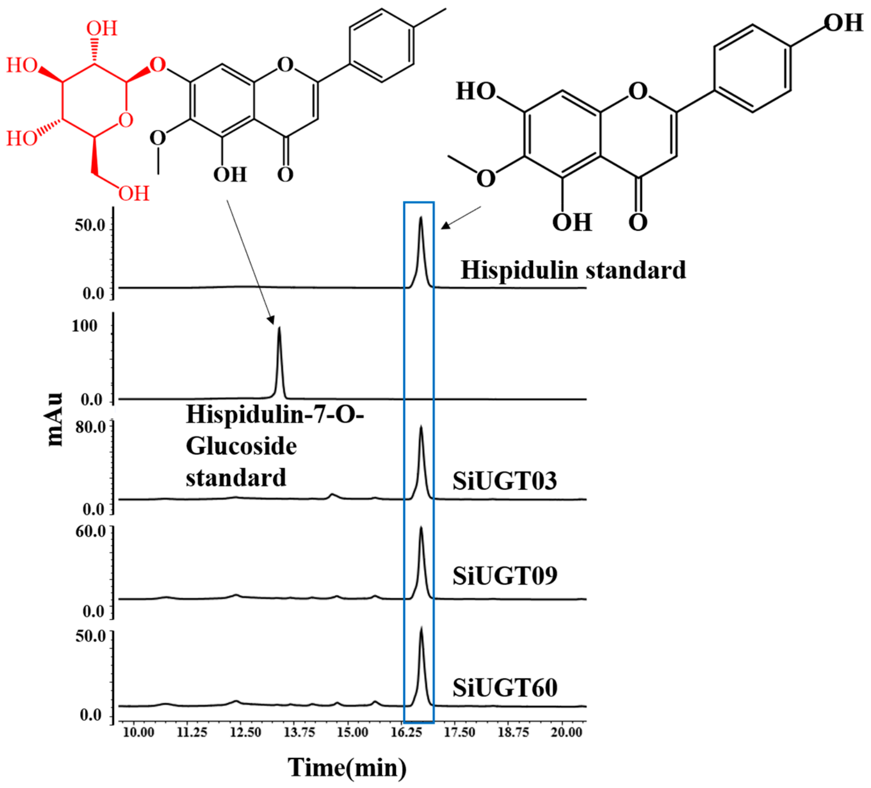

Figure S6. UPLC profiles of in vitro glucosylation reactions of SiUGTs with hispidulin substrate.

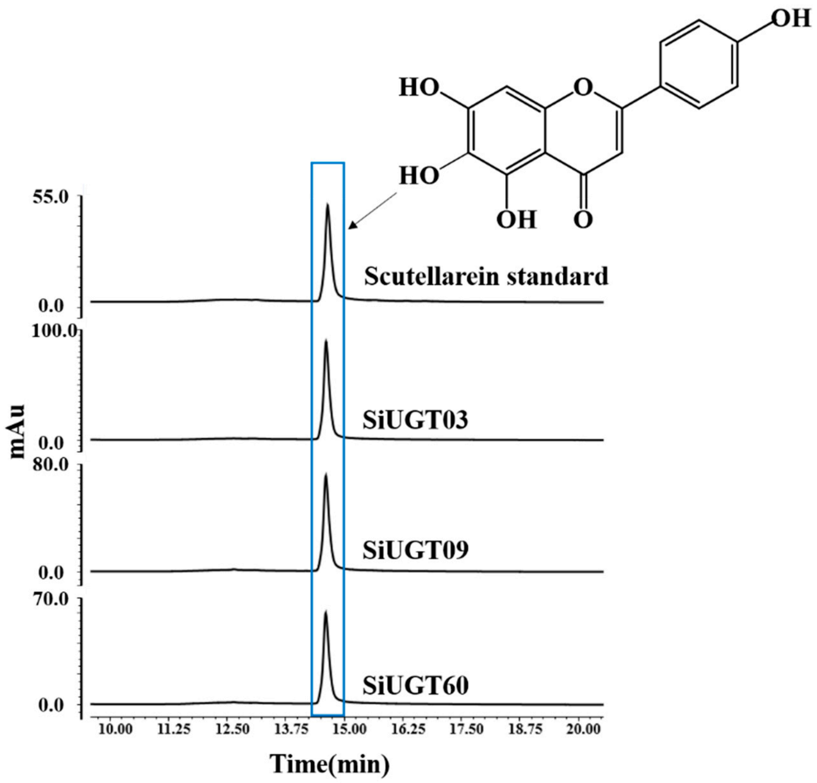

Figure S7. UPLC profiles of in vitro glucosylation reactions of SiUGTs with scutellarein substrate.

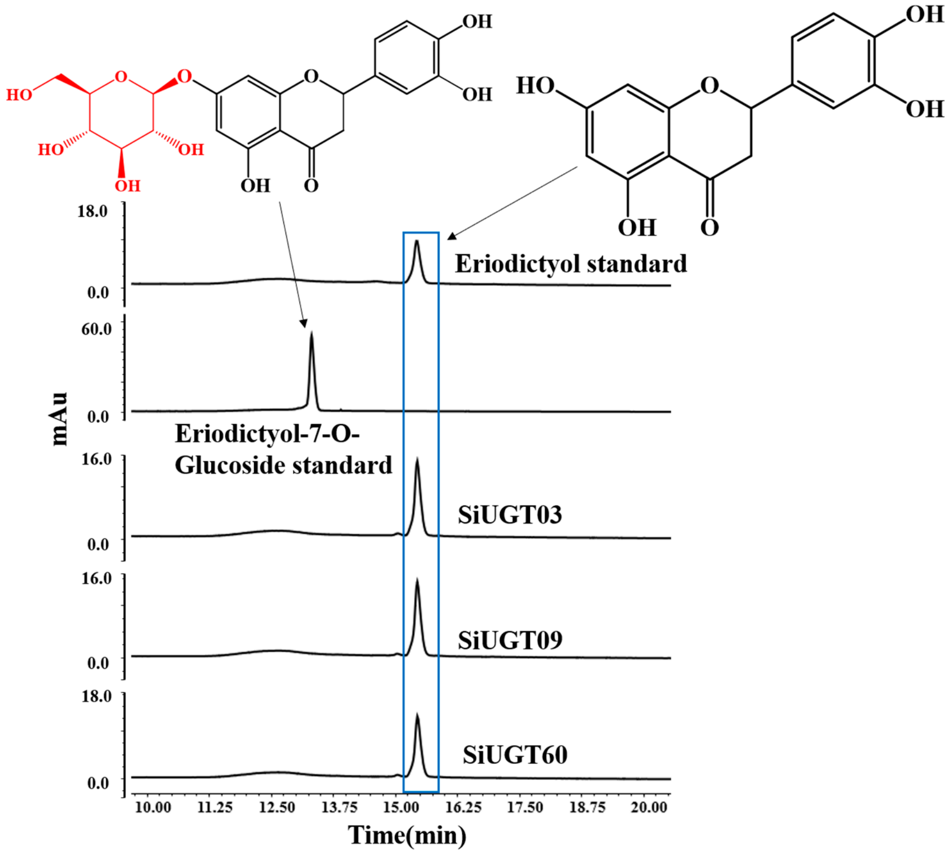

Figure S8. UPLC profiles of in vitro glucosylation reactions of SiUGTs with eriodictyol substrate.

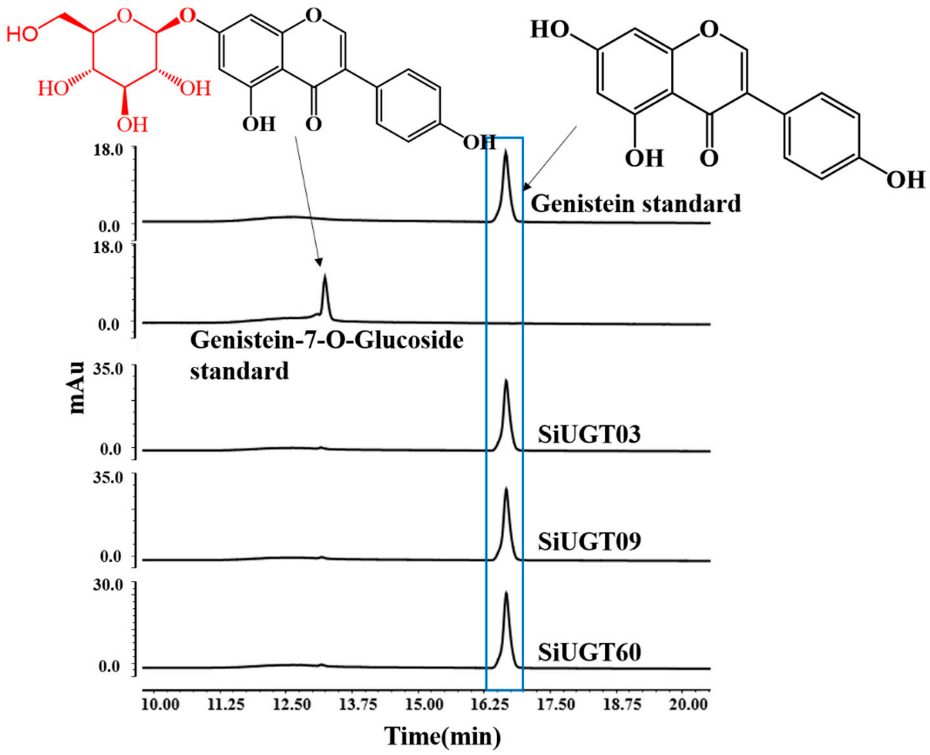

Figure S9. UPLC profiles of in vitro glucosylation reactions of SiUGTs with genistein substrate.

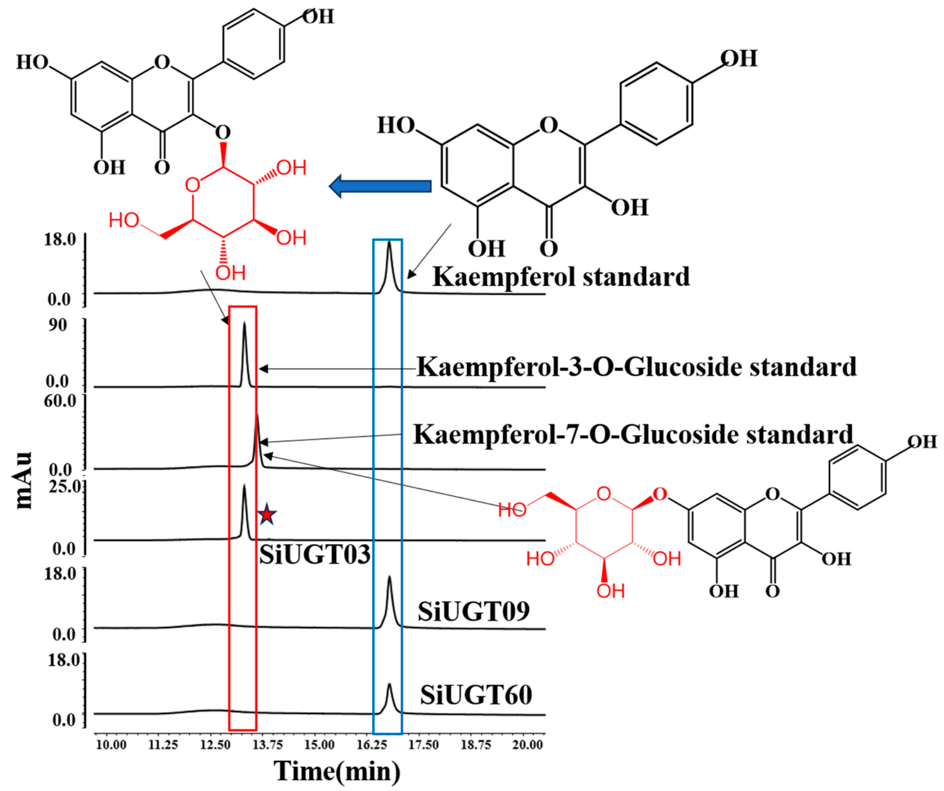

Figure S10. UPLC profiles of in vitro glucosylation reactions of SiUGTs with kaempferol substrate.

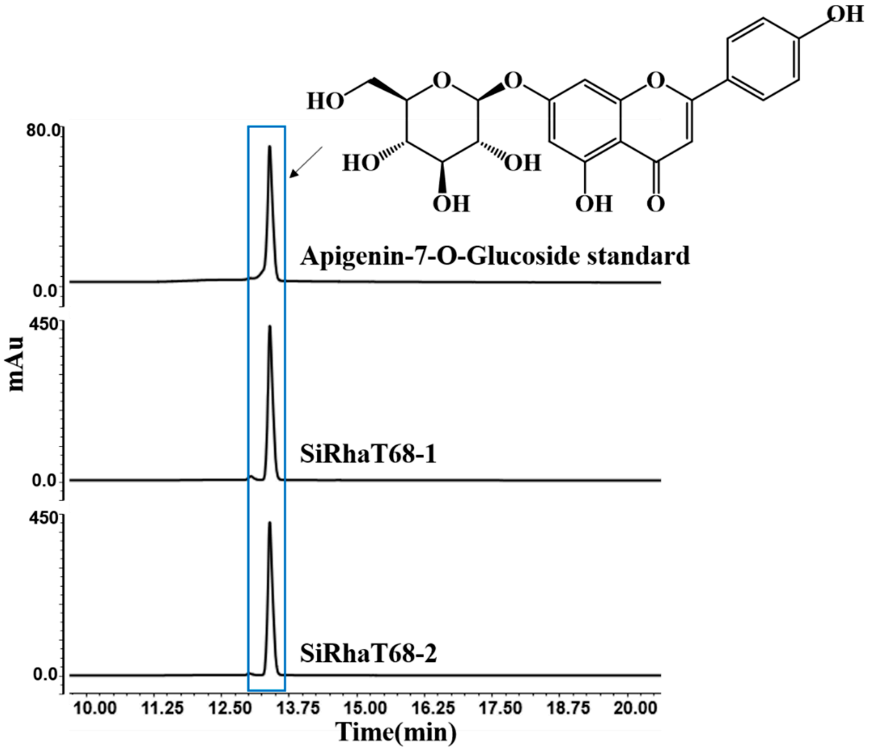

Figure S11. UPLC profiles of in vitro rhamnosylation reactions of SiRhaTs with apigenin-7-glucoside substrate.

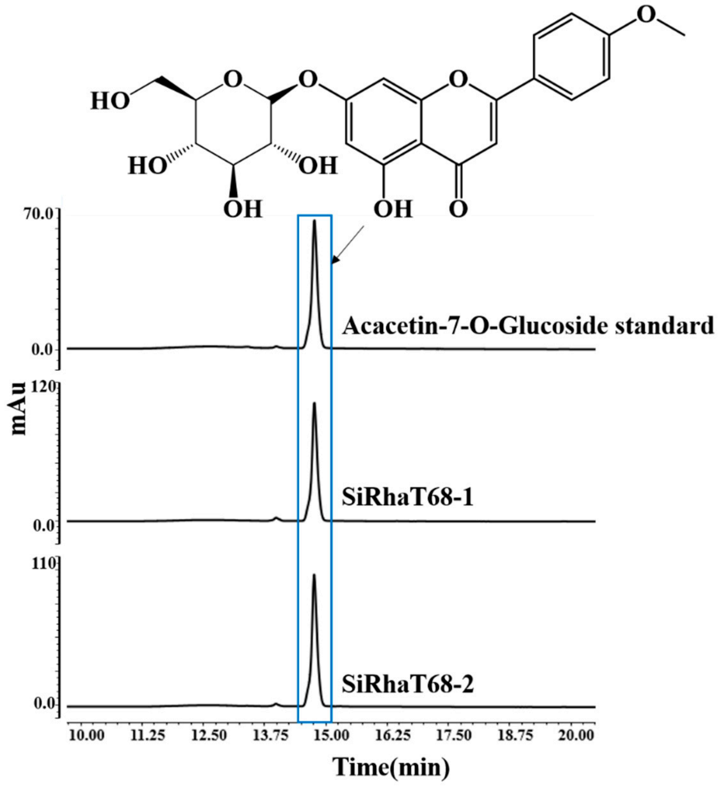

Figure S12. UPLC profiles of in vitro rhamnosylation reactions of SiRhaTs with acacetin-7-glucoside substrate.

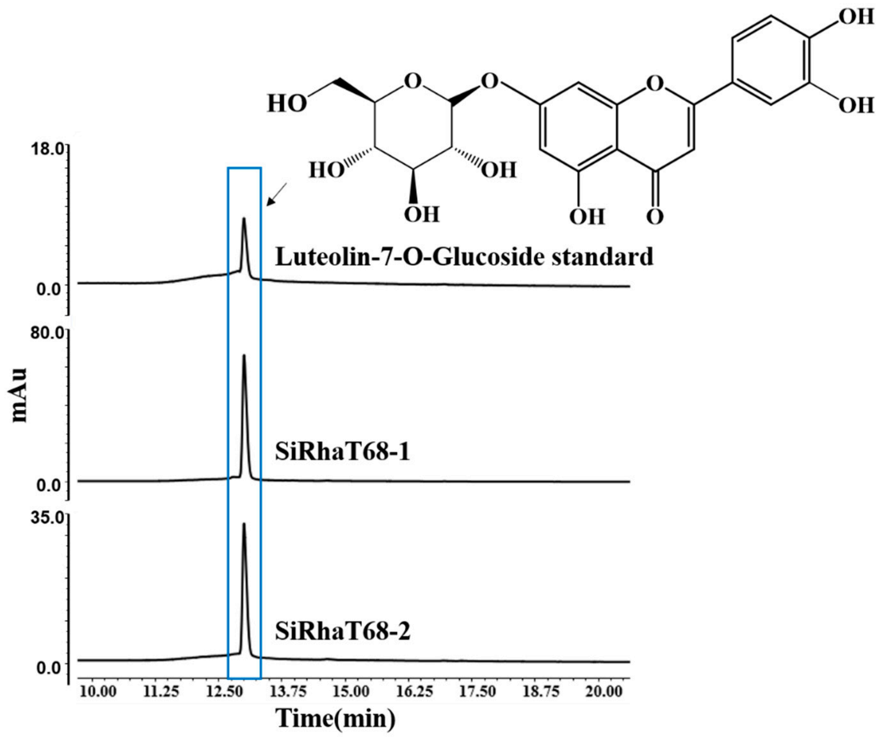

Figure S13. UPLC profiles of in vitro rhamnosylation reactions of SiRhaTs with luteolin-7-glucoside substrate.

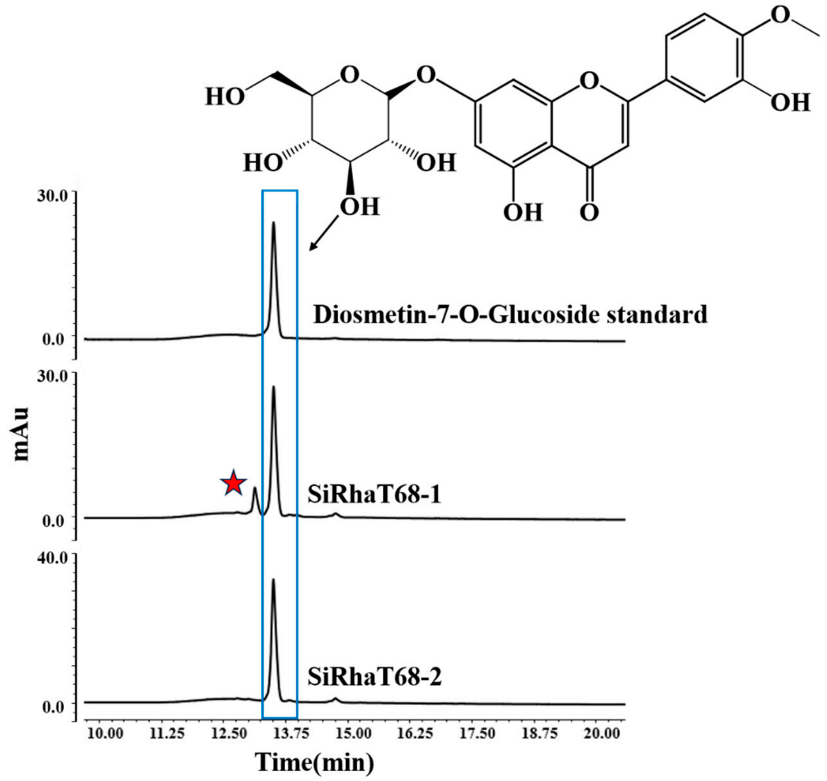

Figure S14. UPLC profiles of in vitro rhamnosylation reactions of SiRhaTs with diosmetin-7-glucoside substrate.

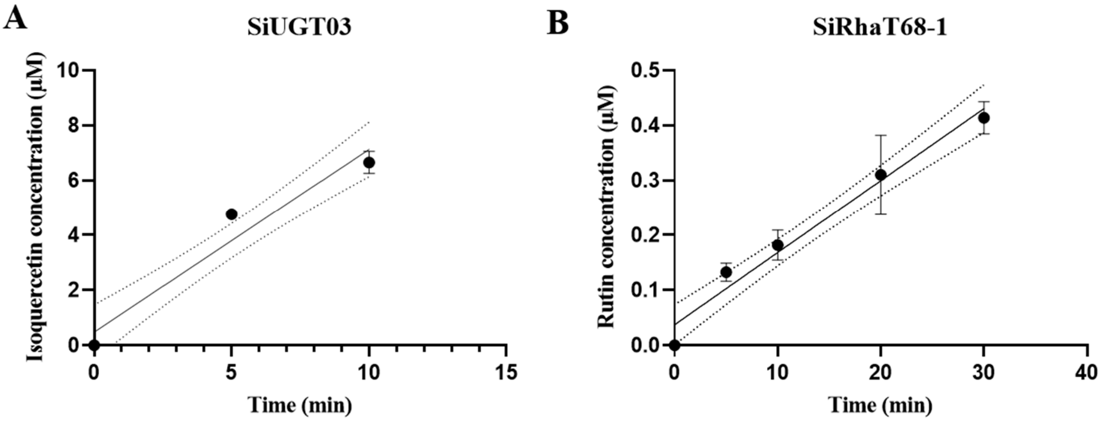

Figure S15. Time-course assays to verify the linear range of product formation for SiUGT03 and SiRhaT68-1. (A) Isoquercetin production catalyzed by SiUGT03, sampled at 0, 5, and 10 min. Linear regression yielded $R^2 = 0.9371$, and substrate consumption was about 6.7% at 10 min (<10%). (B) Rutin production catalyzed by SiRhaT68-1, sampled at 0, 5, 10, 20, and 30 min. Linear regression yielded $R^2 = 0.9516$, indicating linear product formation. Substrate consumption was about 0.51% at 30 min. Data points represent mean values, and error bars indicate SD (n = 3).
